# Establishing the Ethiopian Obstetric Surveillance System for Monitoring Maternal Outcomes in Eastern Ethiopia: A Pilot Study

**DOI:** 10.9745/GHSP-D-22-00281

**Published:** 2023-04-28

**Authors:** Abera Kenay Tura, Sagni Girma, Yadeta Dessie, Delayehu Bekele, Jelle Stekelenburg, Thomas van den Akker, Marian Knight, on behalf of the Ethiopian Obstetric Surveillance System Steering Committee

**Affiliations:** a School of Nursing and Midwifery, College of Health and Medical Sciences, Haramaya University, Harar, Ethiopia.; b Department of Obstetrics and Gynaecology, University Medical Centre Groningen, University of Groningen, the Netherlands.; c Department of Obstetrics and Gynaecology, Leiden University Medical Centre, Leiden, the Netherlands.; d School of Public Health, College of Health and Medical Sciences, Haramaya University, Harar, Ethiopia.; e Department of Obstetrics and Gynaecology, St. Paul’s Hospital Millennium Medical College, Addis Ababa, Ethiopia.; f Department of Health Sciences, Global Health, University Medical Centre Groningen, University of Groningen, the Netherlands.; g Department of Obstetrics and Gynaecology, Leeuwarden Medical Centre, Leeuwarden, the Netherlands.; h Athena Institute, Vrije Universiteit Amsterdam, Amsterdam, the Netherlands.; i National Perinatal Epidemiology Unit, University of Oxford, Oxford, United Kingdom.; j Members listed at the end of the article.

## Abstract

**Background:**

Although the majority of maternal deaths and complications occur in low-resource settings, almost all existing strong registration and confidential enquiry systems are found in high-resource settings. We developed and piloted the Ethiopian Obstetric Surveillance System (EthOSS), based on the successful United Kingdom Obstetric Surveillance System (UKOSS) methodology, in 3 regions in Ethiopia to improve ongoing surveillance and tracking of maternal morbidities and deaths, as well as confidential enquiry, compared to the currently used maternal death surveillance and response program in Ethiopia.

**Methods:**

We launched the EthOSS monthly case notification system in 13 hospitals in eastern Ethiopia in April 2021. Study participants included women admitted to the hospitals from April to September 2021 with major adverse obstetric conditions during pregnancy, childbirth, or within 42 days of termination of pregnancy. Designated clinicians at the hospitals used a simple online system to report the number of cases and maternal deaths monthly to the EthOSS team. We present findings on the incidence and case fatality rates for adverse conditions included in the EthOSS.

**Results:**

Over the 6-month pilot period, 904 women with at least 1 EthOSS condition were included in the study, of whom 10 died (case fatality rate, 1.1%). Almost half (46.6%, 421/904) sustained major obstetric hemorrhage, 38.7% (350/904) severe anemia, 29.5% (267/904) eclampsia, 8.8% (80/904) sepsis, and 2.2% (20/904) uterine rupture. To enable care improvement alongside surveillance, the local committee received training on confidential enquiry into maternal deaths from internal and external experts.

**Conclusions:**

In this facility-based project, data on severe adverse obstetric conditions were captured through voluntary reporting by clinicians. Further analysis is essential to assess the robustness of these data, and confidential enquiry into maternal deaths for specific cases is planned to investigate the appropriateness of care.

## Abstract (precis)

The authors’ pilot of the Ethiopian Obstetric Surveillance System demonstrates the feasibility of establishing similar systems to monitor maternal outcomes in other low-resource settings.

## Teaser

**Key Findings:**

- Implementation of obstetric surveillance through a network of reporting clinicians and implementers is feasible in a setting like Ethiopia.
- The introduction of regional obstetric surveillance combining mortality and morbidity data may help to improve care by providing more robust, rapid, and context-sensitive data collection.

**Key Implications:**

- Establishing obstetric surveillance in low-resource settings requires addressing challenges related to poor medical recordkeeping and reporting, as well as capacity-building among clinicians and policymakers to generate and use research evidence locally.
- Involving practicing clinicians, decision-makers, and implementers from the onset is essential for successful implementation and sustainability, and including morbidity data rather than focusing on deaths alone could enhance their participation.
- Ministries of health, professional societies, and academic institutions should actively participate in implementing nationwide obstetric mortality and morbidity surveillance, which could strengthen the current approaches to maternal and perinatal death surveillance and response followed by these stakeholders.

## BACKGROUND

Although the majority of maternal deaths and complications occur in low-resource settings, almost all existing strong registration and confidential enquiry systems are found in high-resource settings.^1^^,^^2^ Robust obstetric surveillance systems in high-resource settings are generally acknowledged to contribute to improved understanding of maternal ill-health.^3^^–^^5^ As one of the first such systems, the UK Obstetric Surveillance System (UKOSS) was established in 2005 to better understand the pathophysiology and clinical management of severe pregnancy-related disorders in the United Kingdom.^4^ The Netherlands Obstetric Surveillance System followed in 2011 with national estimates of eclampsia and cardiac arrest.^6^^,^^7^

These high-income country surveillance systems are collaborating with the International Network of Obstetric Survey Systems, but low- and middle-income countries have been absent from this collaboration. By adapting the UKOSS methodology, the first pioneer obstetric surveillance system in a setting with a high burden of maternal mortality and morbidity was initiated in 2015 in the Indian state of Assam (IndOSS-Assam), a novelty in a low- and middle-income country.^8^ In a 6-month feasibility study, it was shown that establishing obstetric surveillance was indeed feasible,^8^^,^^9^ and this program has now been expanded to 3 other regions in India.^10^

UKOSS has always worked closely with the United Kingdom’s Confidential Enquiry into Maternal Deaths (CEMD) program. We postulated that directly combining a confidential enquiry into severe maternal outcomes with obstetric surveillance that continuously tracks women with such outcomes may catalyze a process of translating evidence into concrete actions to improve care.^11^ Understanding the pathways leading to severe maternal outcomes, including maternal mortality, will enable options for prevention and early management. Given the continued extremely high burden of pregnancy-related illness in sub-Saharan Africa, it is imperative to establish robust obstetric surveillance combined with effective confidential enquiry.

In late 2014, although Ethiopia introduced the maternal death surveillance and response (MDSR) program as part of the accelerating maternal mortality reduction strategy in accordance with World Health Organization guidance,^12^^,^^13^ the MDSR focused on identifying maternal deaths only. Unfortunately, the MDSR was shown to capture less than 10% of all expected maternal deaths.^14^^,^^15^ It has recently been proposed that health workers’ and managers’ fears of being blamed and shamed as part of the often-identifiable, facility-based MDSR implementation have contributed to this underreporting.^16^

To overcome these challenges, we felt that ongoing surveillance or tracking of maternal morbidities in addition to deaths might be beneficial for several reasons: (1) it may remove some of the sensitivities surrounding reviews of maternal deaths only, (2) it may more robustly and swiftly evaluate the impact of local interventions and facilitate a quick “learning system” at the local level, and (3) it may ultimately facilitate a women-centered approach by including survivor perspectives on maternity care. In addition, we believed that our ultimate aim of arriving at context-specific, facility-based quality improvement required more detailed data, enhanced local ownership, and a closer link to the local professionals through local confidential enquiry than is currently achieved through the MDSR. Moreover, where the MDSR aims to identify all maternal deaths, including those at the community level, we focused on facility-based complications and deaths to enable improved care at the facility level.

With the intention of piloting the feasibility of a regionally tailored obstetric surveillance system combined with CEMD in a low-resource setting in sub-Saharan Africa, the Ethiopian Obstetric Surveillance System (EthOSS) project was initiated in 2020 through a collaboration of Haramaya University, the National Perinatal Epidemiology Unit at the University of Oxford, Leiden University Medical Centre, and the University Medical Centre Groningen. In this article, we present the process of initiating the EthOSS project and preliminary findings from the pilot.

We piloted the Ethiopian Obstetric Surveillance System, a regionally tailored obstetric surveillance system that identified facility-based maternal morbidities and deaths.

## DEVELOPMENT OF THE ETHIOPIAN OBSTETRIC SURVEILLANCE SYSTEM

We implemented the EthOSS by considerably adapting the UKOSS methodology to suit the Ethiopian context for surveillance of major adverse obstetric conditions, as was previously done in India for the IndOSS-Assam.^8^ Details of the UKOSS methodology and its adaptation in Assam have been described elsewhere.^3^^,^^8^ Following the IndOSS-Assam example, and keeping in mind the desired principle to incorporate confidential enquiry into the surveillance system, the initiation of the EthOSS comprised 4 main activities: (1) establish a steering committee, (2) establish priority conditions for reporting, (3) pilot a case notification system in participating hospitals, and (4) implement training on CEMD.

### Establish a Steering Committee

We established a multidisciplinary steering committee to oversee project implementation and prioritize the conditions included in the surveillance platform. Criteria for membership on the committee considered experience, professional mix, regional representation, and representation from all care levels and academia. A letter was sent to the Federal Ministry of Health (MOH), Ethiopian Society of Obstetricians and Gynaecologists, and Ethiopian Midwifery Association asking them to nominate 1 steering committee member.

The 18-member committee included national representatives from the MOH, Ethiopian Society of Obstetricians and Gynaecologists, Ethiopian Midwifery Association, and Professional Association of Emergency Surgery Officers of Ethiopia, as well as local authorities including heads of regional health bureaus in included regions; practitioners (consultant obstetricians, senior midwives, and critical care specialists); and academics. Included among these are 3 EthOSS investigators based at Haramaya University, with the first author serving as secretary.

Over the study period, 3 steering committee meetings were planned to oversee the project implementation. Two meetings were successfully held, and the final meeting is scheduled for later in 2023 at the end of the project period. The EthOSS research team frequently updated the committee chair on the ongoing project implementation through emails and regular progress reports.

### Establish Priority Conditions for Reporting

We selected conditions to be included in the EthOSS surveillance by identifying the major causes of maternal deaths from the annual MDSR reports, combined with suggestions and ratings by representatives from the participating hospitals during the project launch. Attendants of the project launching ceremony included steering committee members, as well as medical directors and chief executive officers of respective hospitals, a senior midwife, and a senior obstetrician-gynecologist. During the launch ceremony, a list of the major adverse obstetric conditions from the MDSR reports and other sources was presented. Attendees were asked to rate which of these conditions should be monitored under the EthOSS consortium—the network of participating hospitals, hospital clinicians, EthOSS investigators, and regional and zonal health bureau heads that work together to attain the EthOSS goals—and in which order of importance.

The rated conditions from the launch meeting were presented at the first steering committee meeting 3 weeks later. Considering burden and case fatality rates, simplicity of identification, and potential for improvement in outcomes, the steering committee then made the final selection of the conditions to include in the surveillance. No specific weighting was applied. The final selection by the steering committee was accepted by the broader EthOSS consortium.

For several years, obstetric hemorrhage, hypertensive disorders of pregnancy, sepsis, and anemia have remained the leading causes of maternal deaths in Ethiopia.^12^^,^^14^^,^^15^ In addition to these conditions, we reported other severe maternal outcomes in eastern Ethiopia to be associated with these conditions.^17^ As such, the steering committee prioritized 5 conditions to be included in the EthOSS surveillance: major obstetric hemorrhage, eclampsia, uterine rupture, maternal sepsis, and severe anemia. We summarize working definitions for each condition in Table 1.

**TABLE 1.** Working Definitions of Obstetric Conditions Included in the Ethiopian Obstetric Surveillance System Pilot Study, April 21–September 21, 2021

**Conditions**	**Definitions**
Obstetric hemorrhage	Excessive bleeding (usually related to pregnancy) in a parturient (includes both antepartum and postpartum hemorrhage)
Antepartum hemorrhage	Bleeding from or into the genital tract, occurring from 28+0 weeks of pregnancy and before the birth of the baby
Postpartum hemorrhage	Excessive bleeding (more than 500 mL for vaginal delivery and 1,000 mL for cesarean delivery) following the birth of a baby
Eclampsia	Diastolic blood pressure ≥90 mm Hg or proteinuria +3 and convulsion or coma^15^
Uterine rupture	Complete rupture of uterus during labor and/or confirmed later by laparotomy^15^
Sepsis	Clinical sign of infection and 3 of the following: temperature >38°C or respiration rate 20/min, pulse rate >90/min, white blood count >12,000^15^
Severe anemia	Hemoglobin <7mg/dl^15^

### Pilot a Case Notification System in Participating Hospitals

**FIGURE 1:**
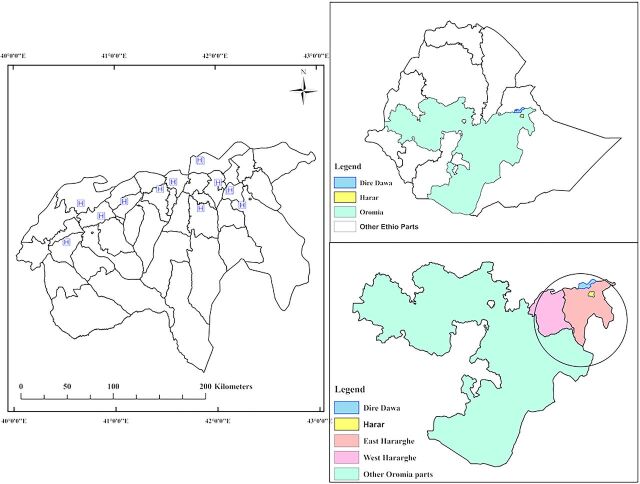
Ethiopian Obstetric Surveillance System Hospital Study Sites Abbreviation: HFSUH, Hiwot Fana Specialized University Hospital.

The decision was made to include all 13 hospitals in eastern Ethiopia in the piloting phase of the EthOSS. Because the region consisted of hospitals ranging from district hospitals located in rural towns to a tertiary academic center in an urban setting, such inclusion was hypothesized to enable the documentation of important lessons pertaining to the referral system between facilities as a prerequisite for nationwide scale-up. The participating hospitals are located in the Harari Region, Dire Dawa City Administration, and 2 zones from Oromia National Regional State (East and West Hararghe Zones) (Figure 1).

We hypothesized that including district hospitals and a tertiary academic center would enable documentation of lessons pertaining to the referral system between facilities as a prerequisite for nationwide scale-up.

**FIGURE 2:**
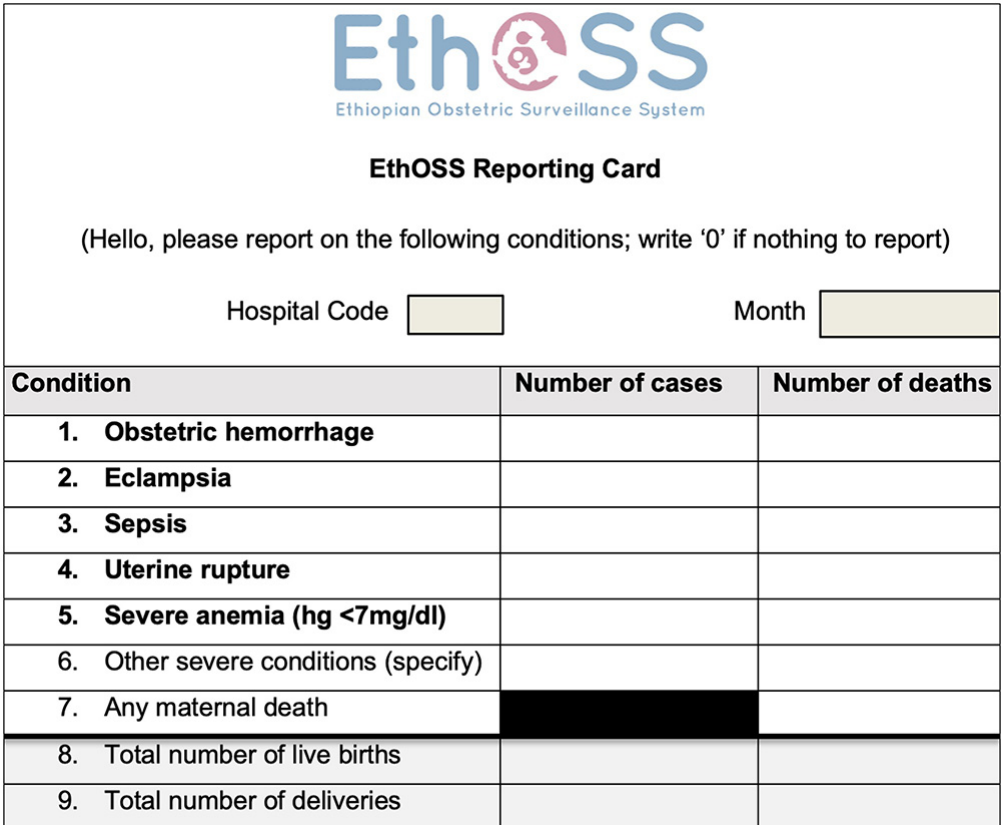
Ethiopian Obstetric Surveillance System Monthly Reporting Card

Under the supervision of the steering committee, we launched the EthOSS monthly case notification system in 13 hospitals in April 2021. To avoid overburdening the reporting clinicians, we employed a simple online case notification form (Figure 2).

Once the designated facility-based EthOSS coordinators sent the number of cases from each condition, EthOSS data collectors were dispatched to respective hospitals to collect more detailed information. Facility-based coordinators were encouraged to register women with any of the EthOSS conditions in the EthOSS logbook daily and report these monthly using the online EthOSS system. When hospital coordinators were away or otherwise not on duty, a replacement coordinator was identified.

Each registry within the EthOSS logbook consists of the patient’s name, EthOSS condition, and medical registration number to facilitate later retrieval of medical records for data collection. The EthOSS research team provided on-site supervision and supported hospital coordinators with any challenges in the implementation process. The overall data reporting was complemented by monthly reminders through phone calls and text messages and quarterly supervisory visits by the EthOSS team.

### Implement Training on CEMD

The final activity was the initiation of CEMD to identify the causes and preventability of maternal deaths and to recommend areas of improvement. We established a CEMD committee that included members with varied professional backgrounds: facility managers, obstetrician-gynecologists, critical care physicians, anesthesiologists, midwives, and emergency obstetric and surgical officers (cadres of associate clinicians).

## METHODS

### Study Participants

All women admitted in public hospitals in eastern Ethiopia—Harari Region, Dire Dawa City Administration, East Hararghe and West Hararghe zones of Oromia Region—constituted the source population for this study. Of these, women who developed obstetric hemorrhage, eclampsia, uterine rupture, sepsis, and severe anemia during pregnancy, childbirth, or within 42 days of termination of pregnancy in hospitals with maternity units from April 2021 to September 2021 comprised the study population.

### Data Collection

Trained EthOSS research assistants based at Haramaya University conducted monthly field visits to respective hospitals to collect detailed data pertaining to reported cases. The research assistants received training from the first investigator on the obstetric surveillance system, objectives of the EthOSS, and common data sources. Data were collected online using the KoBo Toolbox, an open-source suite of tools for data collection and analysis.

From April to September 2021, data were collected on a standard data abstraction sheet adapted from UKOSS studies, national MDSR forms, and other literature.^3^^,^^5^ This sheet contained information on socioeconomic characteristics, obstetric and medical history, and conditions present upon admission, as well as specific interventions and management applied and maternal and perinatal outcomes. The abstraction sheet was divided into 2 parts: general and case-specific conditions. Responses to general questions were recorded for every included case. Case-specific questions collected information for each adverse obstetric condition: obstetric hemorrhage, eclampsia, uterine rupture, sepsis, and severe anemia. Details pertaining to the abstraction sheet, adverse obstetric conditions under surveillance, study protocol, and other project matters will be publicly available on the forthcoming EthOSS website.

### Data Management and Analysis

All collected data were checked for completeness and consistency by the EthOSS investigators before exporting to Stata 13 (StataCorp) for analysis. Summary figures are presented using frequencies and percentages with 95% confidence intervals.

### Ethical Approval

The EthOSS protocol was reviewed and approved by the Institutional Health Research Ethics Review Committee (Ref No. IHRERC/024/2021) of the College of Health and Medical Sciences of Haramaya University, Ethiopia, and the University of Oxford’s Oxford Tropical Research Ethics Committee (OxTREC Reference 530-21). Before data collection, medical directors of respective hospitals and heads of obstetrics and gynecology wards gave their informed consent. While facility identifiers were registered on the EthOSS logbook for tracing cases and other administrative purposes, personal or facility identifiers were not entered into the database.

## RESULTS

### Pilot of Data Registration System

**FIGURE 3:**
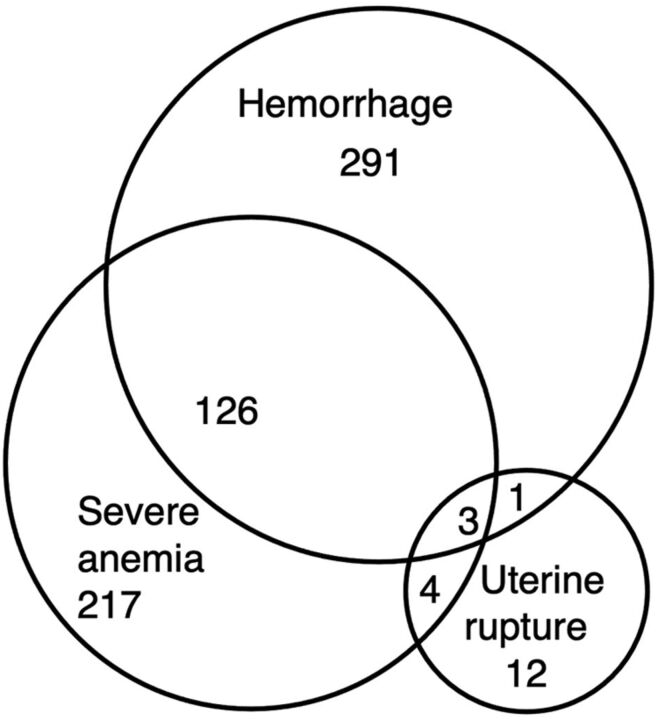
Concurrent Occurrence of Hemorrhage, Uterine Rupture, and Severe Anemia in Eastern Ethiopia

All 13 invited hospitals in the region consented and were included in the EthOSS consortium (Figure 1 and Table 2). From April 1, 2021 to September 30, 2021, a total of 17,317 live births, 904 women with at least 1 EthOSS condition, and 10 maternal deaths were reported through the platform. The most common EthOSS conditions reported were major obstetric hemorrhage (46.6%; 421/904), severe anemia (38.7%; 350/904), and eclampsia (29.5%; 267/904). The overall case fatality rate (CFR) was 1.1%, with sepsis having the highest CFR (2.5%) (Table 3). Although severe anemia was the second most common EthOSS condition reported, more than a third of these cases were also associated with (major or minor) obstetric hemorrhage (Figure 3).

**TABLE 2:** Characteristics of Hospitals in the Ethiopian Obstetric Surveillance System Consortium During the Pilot Study, April 21–September 21, 2021

**Hospital Name**	**Category/Level**	**Location (Town)**	**Most Senior Clinician** ^a^	**Total Live Births^b^**
HFSUH	Tertiary (academic)	Harar	Obstetrician	2,145
Dil Chora	Referral	Dire Dawa	Obstetrician	2,053
Chiro	General	Chiro	Obstetrician	2,152
Gelemso	General	Gelemso	Obstetrician	2,222
Bisidimo	General	Bisidimo	IESO	678
Dedar	General	Dedar	IESO	706
Jugel	General	Harar	Obstetrician	1,098
Garamuleta	General	Girawa	IESO	1,066
Haramaya	General	Haramaya	Obstetrician	2,220
Hirna	Primary	Hirna	IESO	884
Asabot	Primary	Mieso	IESO	401
Sabian	General	Dire Dawa	IESO	1,078
Chelenko	Primary	Chelenko	IESO	614
Total				17,317

Abbreviations: HFSUH, Hiwot Fana Specialized University Hospital; IESO, integrated emergency surgical officer.

^a^ The most senior clinician attending a patient in obstetrics and gynecology.

**TABLE 3.** Distribution of Ethiopian Obstetric Surveillance System Conditions and Maternal Deaths in Eastern Ethiopia During Pilot Study, April 21–September 21, 2021

**Condition**	**Cases, No. (%)** **(N=904)**	**Incidence** ^a^	**Deaths, No.**	**Case Fatality Rate, % (95% CI)**
Obstetric hemorrhage	421 (46.6)	24.3	5	1.2 (0.39, 2.75)
Eclampsia	267 (29.5)	15.4	2	0.8 (0.1, 2.68)
Uterine rupture	20 (2.2)	1.2	0	0.0
Sepsis	80 (8.8)	4.6	2	2.5 (0.3, 8.74)
Severe anemia	350 (38.7)	20.2	1	0.3 (0.01, 1.58)
Total	904^b^ (100)	52.2	10	1.1 (0.53, 2.02)

Abbreviation: CI, confidence interval.

^a^ Per 1,000 live births.

^b^ Actual total exceeds 904 because some women had more than 1 EthOSS condition.

All consenting hospitals successfully returned the monthly EthOSS reporting cards. Some hospital coordinators sent their reports on time, while others required several reminders by phone. Additional on-site supervision by the EthOSS team revealed that, in general, EthOSS conditions were successfully reported, except for sepsis and severe anemia. The supervision visits consisted of meeting with hospital coordinators and reviewing labor, operating room, admission, discharge, and central intensive care unit registers for cross-checking. We observed low reporting from gynecological wards due to the variation in the presence of separate admission and discharge registers. While many hospitals use separate admission and discharge registers in the gynecological ward, the majority of the primary and general hospitals do not have separate gynecological and obstetric registers and instead maintain a single register for both wards. Because many registers are physically located in the obstetrics ward, women with sepsis and severe anemia presenting to the gynecological ward were found to be underreported. Maternal death surveillance, undertaken under the existing maternal and perinatal death surveillance and response program,^18^^,^^19^ includes immediately taking the woman’s medical file to the medical director’s office to prepare for maternal death review and prevent alterations in the documentation of the clinical management. As such, most of the deaths were not captured under the EthOSS platform but instead through our team’s supervision and included accessing the documents kept in medical directors’ offices.

### CEMD Training

As part of reducing maternal deaths from preventable conditions, we initiated CEMD training with a 1-day training provided by experts from the University of Oxford National Perinatal Epidemiology Unit, Leiden University Medical Centre, and Haramaya University College of Health and Medical Sciences. A total of 24 health care providers—obstetricians, critical care physicians and nurses, midwives, and emergency surgical officers—received training on the principles of CEMD, its method of reporting, and other aspects. As part of the training, the trainees conducted reviews of 3 maternal deaths and 1 maternal near miss under supervision. Subsequently, the trainees became members of the EthOSS CEMD committee. After the training, the CEMD committee conducted its first regional chapter meeting to review the maternal deaths reported by the 13 hospitals from April 1 to December 31, 2021. Results will be reported in a subsequent article.

To perform CEMD, the committee assessed cases from all facilities, which were de-identified as part of the CEMD process. In this manner, the introduction of CEMD was not found to overlap with the MDSR program, which has been in place since 2014.^12^ Although both deal with the review of maternal deaths, their approach and purpose differ. The MPDSR is a facility-based review of deaths that occur in the facility or in the community (verbal autopsy) by a multidisciplinary committee to identify the cause of the deaths and design an appropriate response for preventing similar deaths at that facility or in the community in the future. In contrast, CEMD focuses on more general recommendations that come from reviewing deaths that occur in a specific county, region or country.^11^

## DISCUSSION

Having been successfully piloted, the EthOSS is now a second pioneer obstetric surveillance system in low-resource settings, after IndOSS-Assam.^8^ Given that most obstetric surveillance surveys under the auspices of the International Network of Obstetric Survey Systems are conducted in high-income countries,^5^ this implementation of EthOSS affirms that it is also possible to perform continuous tracking at relatively low cost in countries with a higher burden of mortality and morbidity. As many maternal mortality and morbidity estimates originate from projections, successful systems like the EthOSS that present actual reported data can be of paramount importance in the countdown to the Sustainable Development Goal targets for 2030.^20^^,^^21^

The successful implementation of EthOSS affirms that it is possible to perform continuous obstetric surveillance at relatively low cost in countries with a higher burden of mortality and morbidity.

Programs like the EthOSS may help overcome some of the challenges presently identified in the implementation of the MDSR. The inclusion of severe maternal morbidity or maternal near misses, which is more common than maternal mortality, may boost the morale of providers by also generating positive lessons and identifying “great saves.” It may also enable relatively rapid regional data collection necessary for the evaluation of local interventions. Introducing regional CEMD (as opposed to the MDSR by which reviews are conducted at the facility level) may help generate relevant lessons learned by providing a likely truly de-identifiable assessment.^22^^,^^23^ Moreover, the calculations of facility-based case fatality rates might be informative to professionals and policymakers when evaluating which cases may require particular attention.

By scaling up the program to a national system, we hope that the EthOSS will become a powerhouse of data on maternal mortality and morbidity in Ethiopia. Through generating lessons from epidemiological data on maternal morbidity and mortality coupled with robust analysis of causes and preventability of maternal deaths as part of the CEMD, the EthOSS will support the country’s journey to the 2030 targets. By working with the University of Oxford (where both UKOSS and Mothers and Babies: Reducing Risk through Audits and Confidential Enquiries across the UK are based) and the Leiden University Medical Centre (where the Netherlands Obstetric Surveillance System began), the project will deliver significant capacity-building to those involved in the EthOSS consortium.

By scaling up the program to a national system, we hope that the EthOSS will become a powerhouse of data on maternal mortality and morbidity in Ethiopia.

The findings from this study align with the national findings that obstetric hemorrhage, specifically postpartum hemorrhage, is the leading cause of maternal deaths and severe complications.^24^ A 2018 study in 2 of the 13 hospitals in eastern Ethiopia reported obstetric hemorrhage and (severe pre-) eclampsia as the 2 major underlying conditions.^17^ Although anemia is one of the most frequent EthOSS conditions reported in this study, more than a third (36.9%) of the cases of anemia were associated with obstetric hemorrhage. Sepsis and severe anemia might have been underreported, given their reported burden in previous studies. This underreporting might partly be explained by more difficult access to data in gynecological departments.^25^

We believe that the EthOSS platform functioned relatively well by capturing all EthOSS conditions, albeit with likely underreporting of some conditions. In consultation with the hospital managers, we introduced several measures to minimize underreporting, particularly in gynecological wards. Coupled with CEMD, the EthOSS aims to contribute significantly to improving the Ethiopian health system. Identification of health system deficiencies will enable the planning and implementation of health system strengthening and human capacity-building to respond to the persistent high maternal morbidity and mortality. Since Ethiopia, similar to other countries, is planning to initiate a national CEMD to complement the MDSR, lessons from this pilot project may provide essential input into such national policies.^26^ More generally, our findings may feed into efforts to improve the functioning of the MDSR implementation in Ethiopia and beyond. Ultimately, EthOSS could either be integrated into the MDSR approach or function as a complementary system. CEMD enabled closer insight into overarching themes pertaining to cases also included in the MDSR. A similar experience has been reported in Kenya.^26^

### Lessons Learned

Modeled after the UKOSS methodology, the EthOSS platform could be used for monitoring major adverse obstetric conditions elsewhere in Ethiopia and possibly also in other low-resource settings. The simplified report form (Figure 2) minimized the burden on clinicians. The platform may also minimize the fear of blame associated with reporting adverse outcomes because it includes maternal morbidity in addition to maternal mortality. At the same time, through the use of a single unified reporting system, the platform may overcome the problem of professionals needing to respond to multiple requests for reports. Although the platform was reported to be simple and feasible, we believe that fewer EthOSS conditions, mainly sepsis and severe anemia, than expected were reported from gynecological wards. This was likely related to a lack of separate admission and discharge registers in gynecological wards. Such lack of registers was common, especially in primary hospitals. To minimize such underreporting, we supported those hospitals in having separate registers for their gynecological wards. Electronic medical records might overcome such problems.

Steering committee meetings were not held in as timely a manner as planned due to COVID-19 restrictions and security concerns due to civil unrest in the country. The Internet connection was also often unreliable for arranging online meetings. However, we were able to hold the meetings after COVID-19 restrictions were lifted.

Although our pilot shows the feasibility of an integrated surveillance and confidential enquiry system in this low-resource setting, we learned that we were only able to keep coordinators involved voluntarily by providing them with regular feedback, including on-site supervision visits and financial compensation for using Internet data for sending reports. Although we found no reports on the need for financial incentives for reporting clinicians in other settings, it seems essential to regularly negotiate roles and responsibilities and agree on clear terms of reference. We compensated our coordinators for costs incurred when using the Internet to send reports or paying archive room personnel to access patient files, as well as for travel costs for meetings held in Harar and Dire Dawa, which were located far away from their workplace.

Lack of electronic medical records forced us to recruit research assistants for data collection, unlike in the UKOSS process, where designated staff sent case files of each identified case. While the experience in India used research nurses recruited by the project in each hospital for data collection, we recruited research assistants who traveled each month to respective hospitals for data collection, thereby minimizing associated costs.

### Next Steps

Although EthOSS may not capture all maternal mortality and EthOSS conditions—especially those occurring in lower-level facilities or at home—we believe that the majority of women with complications, even those starting labor at home, will end up in facilities. However, given that our primary intention is to improve regional facility-based care, we are also convinced that such improvement in care at the facility level may convince women to come to the facility to give birth. Following similar methodologies as our partners in the International Network of Obstetric Survey Systems, we will examine the extent of completeness of data through capture-recapture methods. Moreover, we are planning to expand the network to lower-level facilities, including health centers and health posts, to broaden the reach of the EthOSS. While the implementation was a success in terms of establishing a network of volunteering clinicians for reporting and capturing facility-based morbidity and mortality—the main measures for this piloting program—the success of EthOSS will be measured by its long-term sustainability and any changes in maternal and child health outcomes. Successful case studies, including their use in developing and revising guidelines and improving women’s survival, will serve as metrics to measure the success of EthOSS in the long term.

The success of EthOSS will be measured by its long-term sustainability and any changes in maternal and child health outcomes.

### Strengths and Limitations

A strength of this study was the capture of both the adverse obstetric conditions and related maternal deaths, which enabled us to calculate case fatality rates of these conditions rather than provide mere descriptions of cases or deaths alone. The use of an anonymous data collection system and the CEMD were thought to reduce underreporting due to fear of being blamed.

The following limitations should be acknowledged. First, incompleteness of medical records and registers hampers the quality of the data and is likely to impact negatively on CEMD effectiveness. Second, our surveillance is a hospital-based system, aiming to improve facility-based maternity care, and therefore misses women with adverse conditions who were treated at the health center or who did not access facility care at all. This might especially underestimate maternal deaths, given that only 58% of maternal deaths occur in hospitals and the remainder in health centers or at home.^24^ Therefore, population-level incidence of adverse obstetric conditions cannot be given.

## CONCLUSIONS

We found that establishing a simple obstetric mortality and morbidity surveillance system integrated with confidential enquiry was feasible in this low-resource setting where the demand for improving obstetric care is high. There is a need for continuous quality improvement initiatives through feedback to the respective hospitals by creating local ownership of the data and by facilitating the translation of such data into relevant clinical improvements.

**EthOSS Steering Committee Members:** Sheleme Humnessa (Federal Ministry of Health, Addis Ababa, Ethiopia; Chairperson); Ibsa Musa (Harari Regional Health Bureau, Harar, Ethiopia), formerly Ibsa Ibrahim (former head of Harari Regional Health Bureau, Harar, Ethiopia); Lemlem Bezabih (Dire Dawa Regional Health Bureau, Dire Dawa, Ethiopia); Abdulaziz Abdurahman (East Hararghe Zone Health Desk, Harar, Ethiopia); Ahmedin Mohamed (West Hararghe Zone Health Desk, Chiro, Ethiopia); Yadeta Dessie (College of Health and Medical Sciences, Harar, Ethiopia; Vice Chair); Roba Ararso (Ethiopian Society of Obstetricians and Gynaecologists, Harar, Ethiopia); Getahun Tiruye (Ethiopian Midwifery Association, Ethiopia); Tadesse Gure (Hiwot Fana Specialized University Hospital, Harar, Ethiopia); Merga Dheresa (College of Health and Medical Sciences, Harar, Ethiopia); Tariku Dingeta (Health, Nutrition and Wellness Theme leader, College of Health and Medical Sciences, Harar, Ethiopia); Redwan Ahmed (College of Health and Medical Sciences, Harar, Ethiopia); Adane Bekele (Chelenko Hospital, Chelenko, Ethiopia), Tolosa Lemi (Gelemso Hospital, Gelemso, Ethiopia); Seble Mengistu (Hiwot Fana Specialized University Hospital, Harar, Ethiopia); Melaku Getachew (Hiwot Fana Specialized University, Harar, Ethiopia); Abera Kenay Tura (College of Health and Medical Sciences, Harar, Ethiopia; Secretary); Sagni Girma (College of Health and Medical Sciences, Harar, Ethiopia).

## Notes

**Peer Reviewed**

**First published online:** March 22, 2023.

## References

[1] Knight M, Nair M, Tuffnell D, Shakespeare J, Kenyon S, Kurinczuk JJ, eds. *Saving Lives, Improving Mothers’ Care - Lessons Learned to Inform Maternity Care From the UK and Ireland Confidential Enquiries Into Maternal Deaths and Morbidity 2013–15*. National Perinatal Epidemiology Unit, University of Oxford; 2017. Accessed February 23, 2023. https://www.hqip.org.uk/wp-content/uploads/2018/02/zliamt.pdf

[2] Hinton L, Locock L, Knight M. Experiences of the quality of care of women with near-miss maternal morbidities in the UK. BJOG. 2014;121(Suppl 4):20–23. 10.1111/1471-0528.12800. 25236629 PMC4312976

[3] Knight M, Kurinczuk JJ, Tuffnell D, Brocklehurst P. The UK Obstetric Surveillance System for rare disorders of pregnancy. BJOG. 2005;112(3):263–265. 10.1111/j.1471-0528.2005.00609.x. 15713136

[4] Knight M, Lindquist A. The UK Obstetric Surveillance System: impact on patient safety. Best Pract Res Clin Obstet Gynaecol. 2013;27(4):621–630. 10.1016/j.bpobgyn.2013.03.002. 23548471

[5] Knight M; INOSS. The International Network of Obstetric Survey Systems (INOSS): benefits of multi-country studies of severe and uncommon maternal morbidities. Acta Obstet Gynecol Scand. 2014;93(2):127–131. 10.1111/aogs.12316. 24382256

[6] Schaap TP, van den Akker T, Zwart JJ, van Roosmalen J, Bloemenkamp KWM. A national surveillance approach to monitor incidence of eclampsia: The Netherlands Obstetric Surveillance System. Acta Obstet Gynecol Scand. 2019;98(3):342–350. 10.1111/aogs.13493. 30346039

[7] Schaap TP, Overtoom E, van den Akker T, Zwart JJ, van Roosmalen J, Bloemenkamp KWM. Maternal cardiac arrest in the Netherlands: a nationwide surveillance study. Eur J Obstet Gynecol Reprod Biol. 2019;237:145–150. 10.1016/j.ejogrb.2019.04.028. 31051417

[8] Nair M, Choudhury MK, Choudhury SS, et al. IndOSS-Assam: investigating the feasibility of introducing a simple maternal morbidity surveillance and research system in Assam, India. BMJ Glob Health. 2016;1(1):e000024. 10.1136/bmjgh-2015-000024. 28588919 PMC5321309

[9] Nair M, Choudhury MK, Choudhury SS, et al. Association between maternal anaemia and pregnancy outcomes: a cohort study in Assam, India. BMJ Glob Health. 2016;1(1):e000026. 10.1136/bmjgh-2015-000026. 28588921 PMC5321311

[10] Nair M, Bezbaruah B, Bora AK, et al. Maternal and perinatal Health Research Collaboration, India (MaatHRI): methodology for establishing a hospital-based research platform in a low and middle income country setting. F1000Res. 2020;9:683. 10.12688/f1000research.24923.3. 33500775 PMC7812614

[11] World Health Organization (WHO). *Beyond the Numbers: Reviewing Maternal Deaths and Complications to Make Pregnancy Safer*. WHO; 2004. Accessed February 23, 2023. https://apps.who.int/iris/handle/10665/42984 10.1093/bmb/ldg009 14711752

[12] Federal Democratic Republic of Ethiopia. Ministry of Health (MOH). *Maternal Death Surveillance and Response (MDSR) Technical Guideline*. MOH; 2014.

[13] World Health Organization (WHO). *Maternal Death Surveillance and Response: Technical Guidance Information for Action to Prevent Maternal Death*. WHO; 2013. Accessed February 23, 2023. https://www.who.int/publications/i/item/9789241506083

[14] Ethiopian Public Health Institute (EPHI). *National MDSR Annual Report 2008 EFY*. EPHI; 2017. Accessed February 23, 2023. https://www.afro.who.int/sites/default/files/2019-02/Ethiopia_MDSR_2008EFY_Annual_Report.pdf

[15] Ethiopian Public Health Institute (EPHI). *National Report on MDSR Data From 2006-2007 EFY*. EPHI; 2016. Accessed February 23, 2023. https://www.afro.who.int/sites/default/files/2019-02/First%20National%20MDSR-Report%202006_2007%20EFY.pdf

[16] Willcox ML, Okello IA, Maidwell-Smith A, Tura AK, van den Akker T, Knight M. Maternal and perinatal death surveillance and response: a systematic review of qualitative studies. Bull World Health Organ. 2023;101(1):62–75G. 10.2471/BLT.22.288703. 36593778 PMC9795385

[17] Tura AK, Zwart J, van Roosmalen J, Stekelenburg J, van den Akker T, Scherjon S. Severe maternal outcomes in eastern Ethiopia: application of the adapted maternal near miss tool. PLoS One. 2018;13(11):e0207350. 10.1371/journal.pone.0207350. 30427926 PMC6235311

[18] Hadush A, Dagnaw F, Getachew T, Bailey PE, Lawley R, Ruano AL. Triangulating data sources for further learning from and about the MDSR in Ethiopia: a cross-sectional review of facility based maternal death data from EmONC assessment and MDSR system. BMC Pregnancy Childbirth. 2020;20(1):206. 10.1186/s12884-020-02899-8. 32272930 PMC7147013

[19] Abebe B, Busza J, Hadush A, et al. ‘We identify, discuss, act and promise to prevent similar deaths’: a qualitative study of Ethiopia’s Maternal Death Surveillance and Response system. BMJ Glob Health. 2017;2(2):e000199. 10.1136/bmjgh-2016-000199. 28589016 PMC5435261

[20] World Health Organization (WHO). *Trends in Maternal Mortality 2000 to 2017: Estimates by WHO, UNICEF*. WHO; 2019.

[21] Alkema L, Chou D, Hogan D, et al. Global, regional, and national levels and trends in maternal mortality between 1990 and 2015, with scenario-based projections to 2030: a systematic analysis by the UN Maternal Mortality Estimation Inter-Agency Group. Lancet. 2016;387(10017):462–474. 10.1016/S0140-6736(15)00838-7. 26584737 PMC5515236

[22] Melberg A, Mirkuzie AH, Sisay TA, Sisay MM, Moland KM. 'Maternal deaths should simply be 0': politicization of maternal death reporting and review processes in Ethiopia. Health Policy Plan. 2019;34(7):492–498. 10.1093/heapol/czz075. 31365076 PMC6788214

[23] Tura AK, Fage SG, Ibrahim AM, et al. Beyond no blame: practical challenges of conducting maternal and perinatal death reviews in eastern Ethiopia. Glob Health Sci Pract. 2020;8(2):150–154. 10.9745/GHSP-D-19-00366. 32461200 PMC7326520

[24] Ethiopian Public Health Institute Public Health Emergency Management Center (PHEM). *National Maternal and Perinatal Death Surveillance and Response (MPDSR) System Annual Report 2012 EFY*. PHEM; 2012. Accessed February 23, 2023. https://www.ephi.gov.et/images/Annual-report-of-MPDSR---final_F_11_22_2020.pdf

[25] Ethiopian Public Health Institute Center for Public Health Emergency Management (PHEM). *National Maternal Death Surveillance and Response (MDSR) Annual Report, 2009 EFY*. PHEM; 2010. Accessed February 23, 2023. https://www.afro.who.int/sites/default/files/2019-02/Ethiopia_MDSR_2009EFY_Annual_Report_0.pdf

[26] Republic of Kenya. Ministry of Health (MOH). *Saving Mothers Lives 2017*. *First Confidential Report Into Maternal Deaths in Kenya*. MOH; 2017. Accessed February 23, 2023. https://familyhealth.go.ke/wp-content/uploads/2018/02/CEMD-Summary-of-findings-Sept-3-FINAL.pdf

